# Role of brain glycogen in the response to hypoxia and in susceptibility to epilepsy

**DOI:** 10.3389/fncel.2015.00431

**Published:** 2015-10-27

**Authors:** Juan C. López-Ramos, Jordi Duran, Agnès Gruart, Joan J. Guinovart, José M. Delgado-García

**Affiliations:** ^1^Division of Neurosciences, Pablo de Olavide UniversitySeville, Spain; ^2^Institute for Research in Biomedicine (IRB Barcelona), The Barcelona Institute of Science and TechnologyBarcelona, Spain; ^3^Centro de Investigación Biomédica en Red de Diabetes y Enfermedades Metabólicas Asociadas (CIBERDEM)Barcelona, Spain; ^4^Department of Biochemistry and Molecular Biology, University of BarcelonaBarcelona, Spain

**Keywords:** brain glycogen, epilepsy, hypobaric hypoxia, kainate, local field potentials, mice

## Abstract

Although glycogen is the only carbohydrate reserve of the brain, its overall contribution to brain functions remains unclear. It has been proposed that glycogen participates in the preservation of such functions during hypoxia. Several reports also describe a relationship between brain glycogen and susceptibility to epilepsy. To address these issues, we used our brain-specific Glycogen Synthase knockout (GYS1^Nestin-KO^) mouse to study the functional consequences of glycogen depletion in the brain under hypoxic conditions and susceptibility to epilepsy. GYS1^Nestin-KO^ mice presented significantly different power spectra of hippocampal local field potentials (LFPs) than controls under hypoxic conditions. In addition, they showed greater excitability than controls for paired-pulse facilitation evoked at the hippocampal CA3–CA1 synapse during experimentally induced hypoxia, thereby suggesting a compensatory switch to presynaptic mechanisms. Furthermore, GYS1^Nestin-KO^ mice showed greater susceptibility to hippocampal seizures and myoclonus following the administration of kainate and/or a brief train stimulation of Schaffer collaterals. We conclude that brain glycogen could play a protective role both in hypoxic situations and in the prevention of brain seizures.

## Introduction

Glycogen is the only storage form of carbohydrate in mammals. Its concentration in the brain is relatively low compared to that found in the most glycogenic tissues, namely liver and skeletal muscle. Within the brain, glycogen is stored mainly in astrocytes (Cataldo and Broadwell, 1986), although neurons also contain a small but functionally important amount (Sáez et al., 2014). Brain glycogen is crucial for neuronal survival and synaptic activity during stress episodes such as hypoglycemia and ischemia (Swanson et al., 1989; Suh et al., 2007), but also in euglycemic conditions during periods of increased brain activity (Shulman et al., 2001; Brown et al., 2005). In this regard, regions of high synaptic activity contain greater glycogen stores (Sagar et al., 1987). Astrocytes need glycogen-derived energy to sustain neuronal function by regulating the concentration of neurotransmitters and ions in the synaptic cleft (Walz, 2000; Tritsch and Bergles, 2007). Thus, inhibition of glycogenolysis raises extracellular glutamate concentrations (Schousboe et al., 2010). In addition, several reports propose that astrocytes metabolize glycogen to lactate, which is then supplied to neurons for oxidative metabolism, in what is known as the astrocyte-neuron lactate shuttle hypothesis (Pellerin et al., 1998). Accordingly, glycogen-derived lactate has been shown to be important for memory formation (Suzuki et al., 2011). Therefore, astrocytic glycogen plays a key role in supporting neurotransmission. We previously described electrophysiological alterations and impairment of memory formation in a mouse model in which muscle glycogen synthase (GYS1), the enzyme responsible for the production of glycogen, is specifically deleted from the brain (GYS1^Nestin-KO^; Duran et al., 2013).

Epilepsy is a sudden synchronization of the activity of a group of neurons. Its origins are not completely understood (Dudek et al., 1999), but it is believed to be caused by imbalance between glutamatergic (excitatory) and GABAergic (inhibitory) neurotransmission. During epileptic seizures, both neurons and astrocytes show very high activity, thus increasing energy consumption dramatically. In this scenario, the availability of glycogen could be of great importance, and high glycogen content has been found in biopsies from the brains of epileptic patients (Dalsgaard et al., 2007). Moreover, in the methionine sulfoximine epileptic model, an increase in brain glycogen content has been reported (Phelps, 1975; Hevor et al., 1985), and susceptibility of two inbred mouse strains to methionine sulfoximine inversely correlates with their capacity to accumulate glycogen in the brain (Bernard-Helary et al., 2000). Furthermore, the amount of glycogen in epileptic foci is reduced after seizures induced by kainate (Walls et al., 2014), bicuculline (Folbergrova et al., 1981), homocysteic acid (Folbergrova et al., 2003), pentylenetetrazole (Ingvar et al., 1984), fluorothyl (Folbergrova et al., 1985), 3-mercaptopropionic acid (Folbergrova, 1977), ischemia (Katsura et al., 1994), hypoglycemia (Abdelmalik et al., 2007), maximal electroshock (McCandless et al., 1979), and audiogenic seizures (Schreiber and Ungar, 1984). To analyze the relevance of brain glycogen in epilepsy, here we studied the effects of kainate, a widely used epileptogenic drug (Sperk, 1994), in control and GYS1^Nestin-KO^ animals.

Hypobaric hypoxia is used in animal models to simulate high-altitude conditions that induce impairments in cognitive performance in humans, especially in spatial learning and memory (Shukitt-Hale et al., 1994, 1996; López-Ramos et al., 2007). It has been proposed that brain glycogen is important in metabolically challenging states such as hypoxia. Indeed, exposure to hypobaric hypoxia causes a decrease in brain glycogen (Harik et al., 1995). Furthermore, after a hypoxic period, brain glycogen reaches higher levels than before the insult, a process known as supercompensation. To study the role of the glycogen reservoir in the response to hypobaric hypoxia, we also analyzed the effects of hypobaric hypoxic conditions in control and GYS1^Nestin-KO^ animals.

The present results partly confirmed the above contentions. Thus, GYS1^Nestin-KO^ mice presented larger power spectra of hippocampal local field potentials (LFPs) than controls under hypoxic conditions. KO mice also showed greater excitability than controls for paired-pulse facilitation evoked at the hippocampal CA3-CA1 synapse and greater susceptibility to hippocampal seizures following the administration of kainate and/or a brief train stimulation of Schaffer collaterals. Taken together, our results reveal the relevant role played by glycogen in brain metabolism.

## Materials and Methods

### Animal Studies

GYS1^Nestin-KO^ mice were generated as previously described (Duran et al., 2013). Mice were allowed free access to a standard chow diet and water and maintained on a 12-h/12-h light/dark cycle under specific pathogen-free conditions in the Animal Research Center at the Barcelona Science Park. After weaning the animals at 3 weeks of age, tail clippings were taken for genotyping by PCR. For the electrophysiological and hypobaric-chamber experiments, the mice were transferred to the Animal House of the Pablo de Olavide University (Seville, Spain). Five-month-old male mice were used for these experiments. Animals were kept in collective cages under the conditions described above until surgery. Afterward, they were housed in individual cages until the end of the experiments. All experiments were carried out during the light cycle and following the current European Union Council (2010/63/UE) and Spanish (RD 53/2013) guidelines for the use of laboratory animals in chronic studies. All experimental protocols were also approved by the Ethics Committee (2011/01/11) of the Pablo de Olavide University. ARRIVE guidelines were followed for the reported data.

### Surgery

**FIGURE 1:**
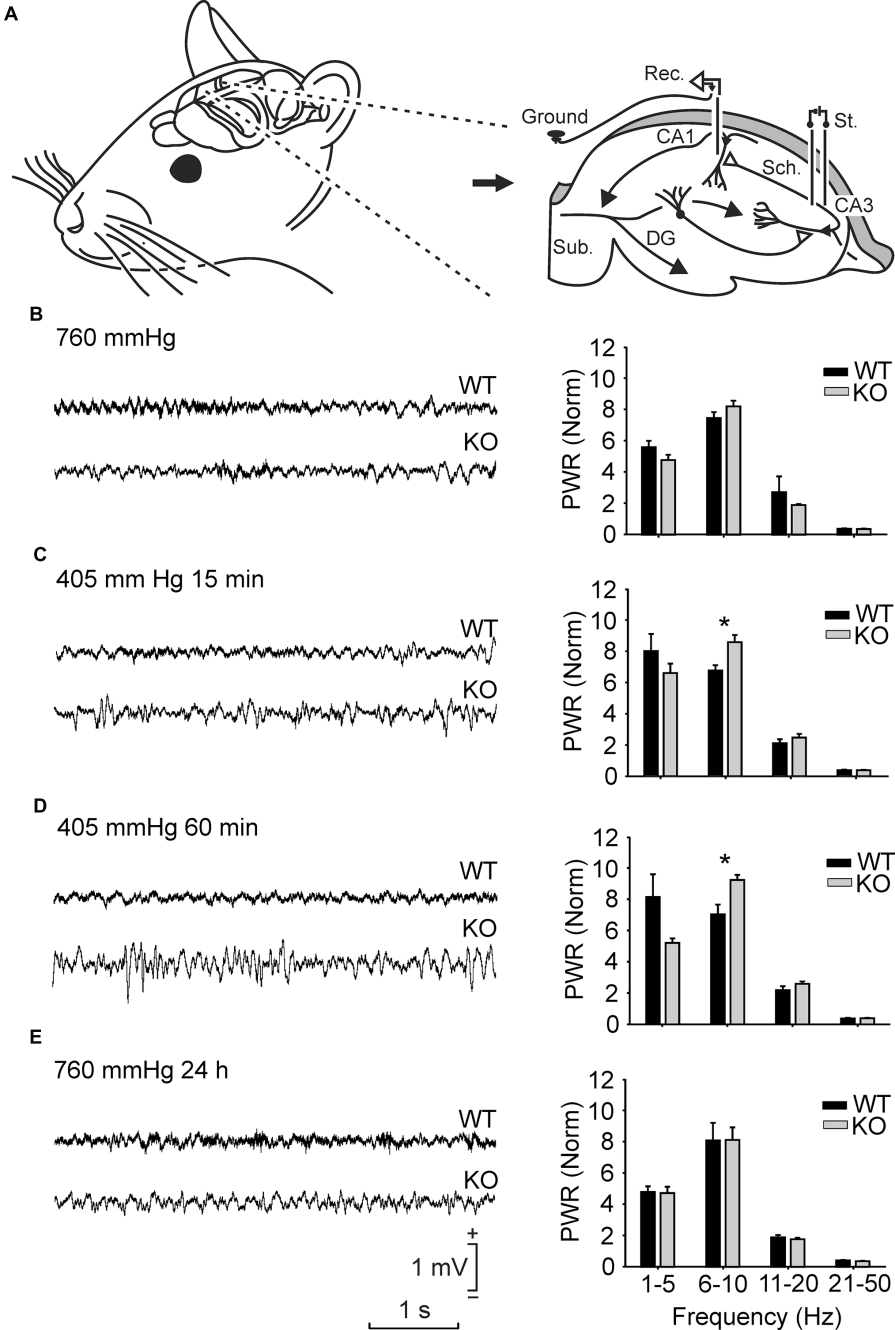
**Spectral analysis of local field potentials (LFPs) recorded in the pyramidal CA1 area from the two groups of mice in different hypobaric situations. (A)** Experimental design. Animals were implanted with recording (Rec.) electrodes in the hippocampal CA1 area and with stimulating (St.) electrodes in the ipsilateral Schaffer collateral-commissural pathway. **(B,E)** From top to bottom are illustrated LFPs recorded and sequenced power spectra (1–5 Hz, 6–10 Hz, 11–20 Hz, and 21–50 Hz) from representative wild-type (WT) and GYS1^Nestin-KO^ (KO) mice placed at ground level (35 m ≈ 760 mmHg) **(B)**, 15 min **(C)** and 60 min **(D)** after being placed under hypobaric conditions (5000 m ≈ 405 mmHg), and 24 h after being returned to ground level (760 mmHg; 24 h) **(E)**. Note the different LFP profiles presented by the two groups of mice in the four different hypobaric situations. Calibrations in **(E)** are also for **(B–D)**. Spectral analysis was averaged from LFPs recorded from *n* ≥ 10 animals per group. Note the higher spectral powers computed from KO mice in the 6–10 Hz band for the two hypobaric situations when compared with those presented by their littermate controls. Amplitudes of power spectra were normalized taking the sum of the total power spectra (from 1 to 50 Hz) of each animal during the initial 760 mmHg situation as a total power (PWR) of 100. Values are expressed as mean ± SEM. Statistical differences, ^∗^*P* < 0.05. Student *t*-test.

Animals were anesthetized with 0.8–3% halothane delivered through a calibrated Fluotec 5 (Fluotec-Ohmeda, Tewksbury, MA, USA) vaporizer at a flow rate of 1–2 L/min oxygen. Following stereotaxic coordinates collected from the Paxinos and Franklin atlas (Paxinos and Franklin, 2001), we implanted bipolar stimulating electrodes in the right Schaffer collateral-commissural pathway of the dorsal hippocampus (2 mm lateral and 1.5 mm posterior to bregma; depth from brain surface, 1.0–1.5 mm) and a recording electrode in the ipsilateral CA1 area (1.2 mm lateral and 2.2 mm posterior to bregma; depth from brain surface, 1.0–1.5 mm). Electrodes were made from 50-μm Teflon-coated tungsten wire (Advent Research Materials Ltd., Eynsham, England). The final location of the recording electrode was determined using as a guide the field potential depth profile evoked by paired (40 ms of interval) pulses presented to the Schaffer collateral pathway. A bare silver wire (0.1 mm) was affixed to the skull as ground. The four wires were connected to a 4-pin socket and the latter was fixed to the skull with the help of two small screws and dental cement. Further details of this chronic preparation have been reported elsewhere (see **Figure 1A**; Madroñal et al., 2009; Gruart et al., 2012).

### LFP Recordings

For LFP recordings, animals were placed in individual (15 cm × 5 cm × 12 cm) methacrylate cages. After a brief period of exploration, before the beginning of the recordings, animals remained in awaked immobility, avoiding behavior-dependent changes in the responses (Cao and Leung, 1991). Recordings were carried out for 5 min. The power spectrum of hippocampal LFP activity was calculated with the help of the MatLab 7.4.0 software (MathWorks, Natick, MA, USA), using the fast Fourier transform with a Hanning window, expressed as relative power and averaged across each recording session (Madroñal et al., 2009).

### Input/Output Curves and Paired-pulse Facilitation in Behaving Mice

We also recorded monosynaptic field excitatory postsynaptic potentials (fEPSPs) evoked in the hippocampal CA1 area by the electrical stimulation of ipsilateral Schaffer collaterals. In accordance with previous electrophysiological studies in alert behaving mice (Madroñal et al., 2009; Gruart et al., 2012), for input/output curves, mice were stimulated at CA3–CA1 synapses with paired pulses (40 ms of inter-pulse interval) of increasing (0.02–0.2 mA) intensity. In addition, the effect of paired pulses at various inter-pulse intervals (10–500 ms) was checked. For this, we used intensities corresponding to ≈40% of the amount required to evoke a saturating response (i.e., when responses reach maximum amplitudes). In all cases, pairs of pulses of a given intensity were repeated ≥5 times with time intervals ≥30 s, to minimize interference with slower short-term potentiation (augmentation) or depression processes (Madroñal et al., 2009). Moreover, to avoid any cumulative effect, intensities and intervals were presented at random.

### Kainate Injection and Recording of Seizure Activities

To study the propensity of control and KO mice to have convulsive seizures, animals were injected (i.p.) with the α-amino-3-hydroxy-5-methyl-4-isoxazolepropionic acid (AMPA)/kainate-receptor agonist kainic acid (8 mg/kg; Sigma, Saint Louis, MO, USA) dissolved in 0.1 M phosphate-buffered saline (PBS) pH = 7.4. The LFP activity of the pyramidal CA1 area was recorded for 2 h after the injection. Animals were presented with a stimulus session (five 200 Hz, 100 ms trains of pulses at a rate of 1/s) 1 h after the injection (Rangel et al., 2009; Valles-Ortega et al., 2011).

### Experiments Performed in the Hypobaric Chamber

For recordings carried out under hypobaric conditions, the animal was in its home box (15 cm × 5 cm × 12 cm), which was placed inside a home-made cylindrical hypobaric chamber (85 cm high × 80 cm in diameter). The top of the chamber was provided with a transparent methacrylate window to allow external control of the animal’s state. Although the chamber served as a sound attenuator, it was also provided with the white noise (45 dB) produced by the air-renewal system. The recording room was dimly illuminated, avoiding projection of the dim light directly through the window of the hypobaric chamber. The desired simulated altitude (5000 m, 405 mmHg, PO_2_ 85.05) was achieved in ≈15 min by means of the progressive extraction of air from the chamber through a vacuum pump (SB.6, D.V.P. Vacuum Technology, Italy) while a minor flux of ambient air was passively introduced inside, controlled by a monitored rotameter. Thus, the pressure inside the chamber decreased, but air-renewal was guaranteed. Animals were maintained at this altitude for a total of 60 min (see Guerra-Narbona et al., 2013, for details).

We recorded LFPs for spectral analysis and fEPSPs for input/output curves at various intensities and inter-pulse intervals before exposure to hypobaric conditions, 15 and 60 min from the beginning of the hypobaric exposure (405 mmHg ≈ 5000 m), and 24 h after.

### Histology

Once the experiments were finished, mice were deeply re-anesthetized (sodium pentobarbital, 50 mg/kg) and perfused transcardially with saline and 4% phosphate-buffered paraformaldehyde. Their brains were removed, postfixed overnight at 4°C, and cryoprotected in 30% sucrose in PBS. Sections of 50 μm were obtained in a microtome (Leica, Germany). Selected sections including the dorsal hippocampus were mounted on gelatinized glass slides and stained with 0.1% toluidine blue to confirm the correct location of stimulating and recording electrodes.

### Data Collection and Analysis

Local field potentials, fEPSPs, and 1-volt rectangular pulses corresponding to kainate injections and brain stimulations were stored digitally on a computer through an analog/digital converter (CED 1401 Plus, Cambridge, England). Data were analyzed off-line: for LFP with the MatLab 7.4.0 software, and for fEPSP recordings with the Spike 2 (CED) program. Five successive fEPSPs were averaged, and the mean value of the amplitude (in mV) was determined. Computed results were processed for statistical analysis using IBM SPSS Statistics 18.0 (IBM, New York, NY, USA) or Sigma Plot 11.0 (Systat Software, Germany). The statistical significance of differences between groups was inferred by one-way ANOVA and ANOVA for repeated measures (data by groups), with a contrast analysis (Dunnett’s posttest) for a further study of significant differences, or the Student *t*-test. Statistical significance was set at *P* < 0.05. Unless other way indicated, mean values are followed by their SEM.

## Results

### Effects of Hypoxia on Control and GYS1^Nestin-KO^ Mice

To analyze the effects of hypobaric hypoxia on the electrophysiological properties of hippocampal circuits in control and GYS1^Nestin-KO^ mice, spontaneous LFPs in the hippocampal CA1 area of the animal resting in its home box were recorded at ground level (760 mmHg ≈ 35 m) and under hypobaric conditions (405 mmHg ≈ 5000 m; **Figures 1B–E**). An initial number of 14 Control and 10 GYS1^Nestin-KO^ mice were used. Exposure for 15 or 60 min to the latter did not evoke any significant change in hippocampal LFP activity in either group, reaching maximum spectral power in the frequency band of 6–10 Hz, ranging between 7.4 ± 0.3 in WT and 8.2 ± 0.3 in GYS1^Nestin-KO^ mice. However, the spectral power of LFPs in GYS1^Nestin-KO^ were significantly (*P* ≤ 0.05) larger than those recorded in the WT group, during the 15 and 60 min hypobaric conditions, in the frequency band of 6–10 Hz (**Figures 1C,D**), due to the decrease of WT values (6.8 ± 0.3 and 7.0 ± 0.6, respectively) and the increase of GYS1^Nestin-KO^ ones (8.6 ± 0.4 and 9.2 ± 0.3, respectively). This observation indicates a higher synchronization of hippocampal LFPs in GYS1^Nestin-KO^ mice under hypoxic conditions.

**FIGURE 2:**
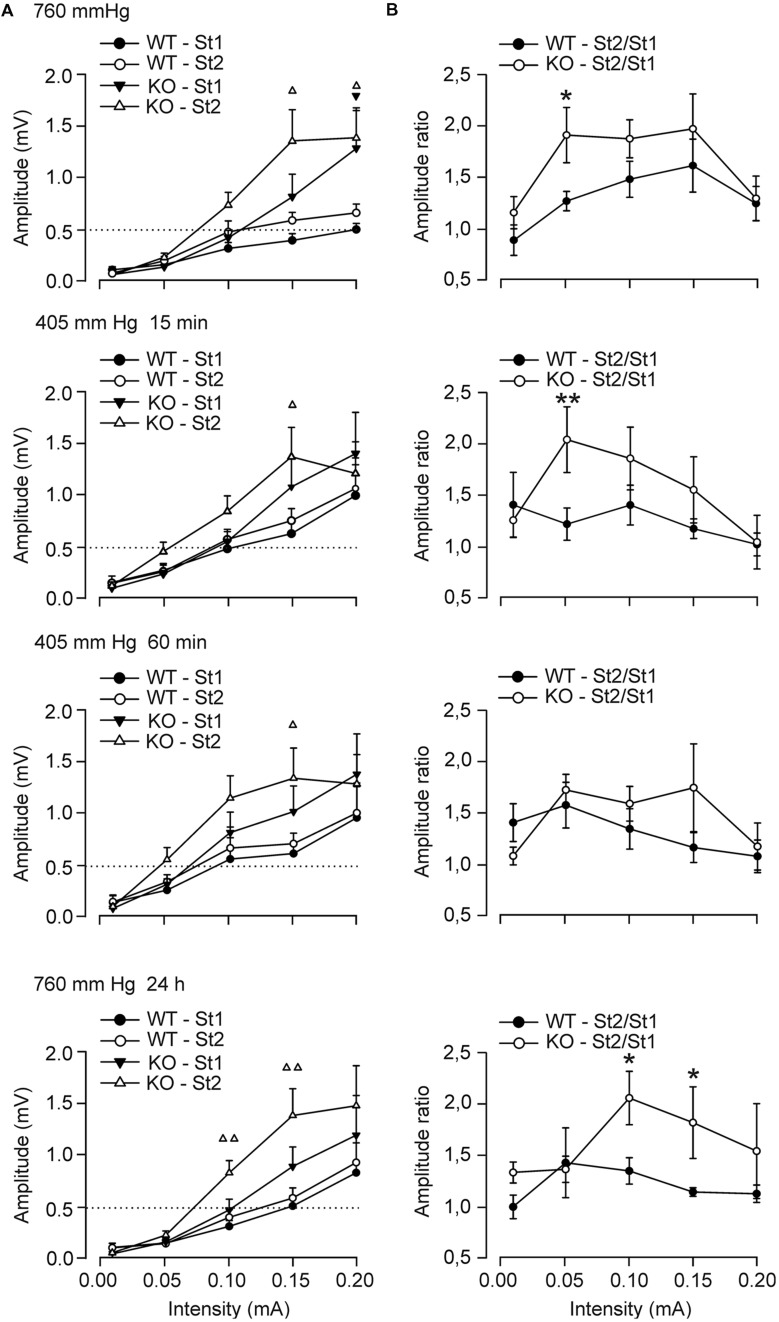
**Electrophysiological changes of hippocampal synapses in *GYS1*^Nestin-KO^ behaving mice under hypobaric conditions. (A)** Input/output curves of fEPSPs evoked by paired pulses (40 ms of inter-stimulus interval) of increasing intensities (0.02–0.2 mA) in wild-type (**A**, WT; *n* = 8) and GYS1^Nestin-KO^ (**B**, KO; *n* = 10) mice. The first and the second fEPSPs are represented. Note that the hypobaric situation increased fEPSP amplitudes, mainly in controls and in *GYS1*^Nestin-KO^ mice for fEPSPs evoked by the second pulse at lower intensities. Dotted lines were set at the maximum values reached by WT animals under the initial ground-level conditions (≈0.5 mV). **(B)** St2/St1 ratios of the fEPSPs showed in **(A)**. The initial significant differences between ratios were abolished after 60 m of hypobaric exposure, and recovered 24 h after it, at higher intensities. Values are expressed as mean ± SEM. Triangles in **(A)** represent differences between firsts (black) and second (white) fEPSPs, *P* < 0.05. Differences between ratios, ^∗^*P* < 0.05; ^∗∗^*P* < 0.01. Student *t*-test.

We next analyzed the response of CA1 pyramidal neurons to paired pulses of increasing intensity presented to the ipsilateral Schaffer collaterals (**Figures 2A,B**). For ground-level conditions (top graphs), the two groups of mice presented an increase in the amplitude of fEPSPs evoked at the CA3–CA1 synapse in parallel with the increased intensity of the pulse applied, with significant facilitation to the second pulses at higher intensities. However, the excitability of the CA3–CA1 synapse differed between groups, with the GYS1^Nestin-KO^ animals being more excitable than the control ones (**Figure 2A**). Thus, Control animals reached amplitudes of 0.49 ± 0.05 mV and 0.65 ± 0.08 mV at higher intensities for the first and second stimulus. In contrast, GYS1^Nestin-KO^ mice reached 1.27 ± 0.3 mV and 1.38 ± 0.2 mV for the first and second stimulus. Exposure to hypobaric conditions caused an increase in the excitability of the CA3–CA1 synapse in both groups, as shown by a leftward displacement of the input/output curves. Although hypoxia increased the excitability in control mice at higher intensities, this was not observed in GYS1^Nestin-KO^, reaching maximum amplitudes after 60 min of hypobaric exposure (0.95 ± 0.3 mV and 1.0 ± 0.2 mV at higher intensities for the first and second stimulus for Control mice, and 1.38 ± 0.3 mV and 1.28 ± 0.2 mV for the first and second stimulus, respectively, for GYS1^Nestin-KO^ animals (**Figure 2A**). Moreover, the differences detected between the St2/St1 ratios before the hypobaric exposure (1.27 ± 0.09 for Control vs 1.91 ± 0.26 for GYS1^Nestin-KO^ mice, at 0.05 mA of intensity), and 15 min after its start (1.22 ± 0.15 for Control vs. 2.05 ± 0.32 for GYS1^Nestin-KO^ mice, at 0.05 mA of intensity) were abolished after 60 min of hypoxia, recovering 24 h later, although at higher intensities (0.1 mA), reaching ratios of 1.35 ± 0.12 for Control vs. 2.06 ± 0.26 for GYS1^Nestin-KO^ mice (**Figure 2B**).

**FIGURE 3:**
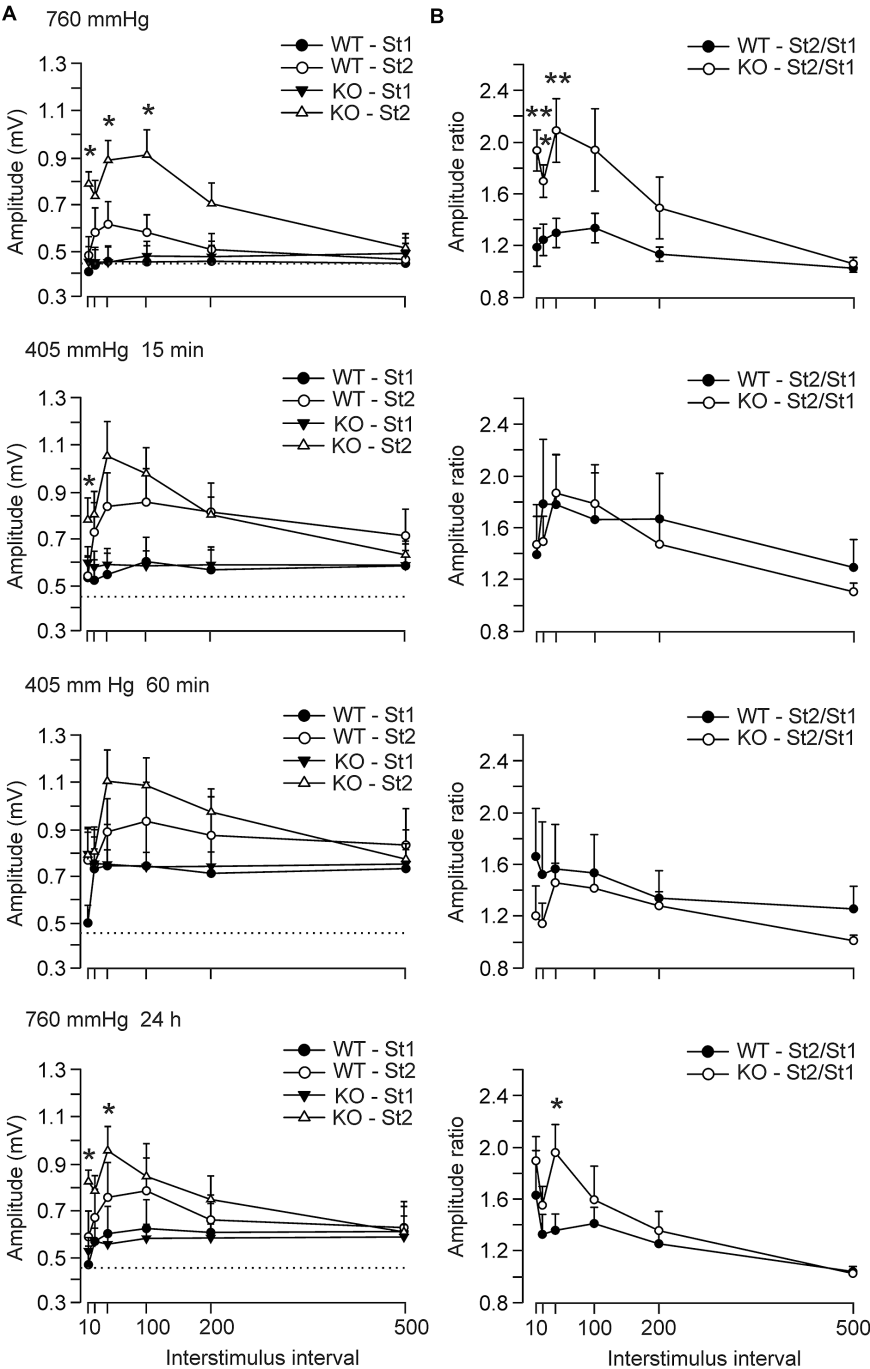
**Electrophysiological changes of hippocampal synapses in *GYS1*^Nestin-KO^ behaving mice under hypobaric conditions. (A)** Paired-pulse facilitation of fEPSPs evoked by a pair of pulses at ≈40% of the maximum intensity and at different inter-pulse intervals (10, 20, 40, 100, 200, and 500 ms) in wild-type (WT; *n* = 12) and GYS1^Nestin-KO^ (KO; *n* = 10) mice. The amplitudes of fEPSPs evoked by the first pulse and the second are represented for the two groups of mice. Note that GYS1^Nestin-KO^ mice presented a larger facilitation (^∗^) to the second pulse for short intervals (10–100 ms) at ground level than did controls, and that the hypobaric conditions evoked a noticeable increase in the amplitude of fEPSPs evoked by the two pulses, although abolished the significant differences between groups. Dotted lines were set at the maximum values reached by WT animals under the initial ground-level conditions (≈0.45 mV). **(B)** St2/St1 ratios of the fEPSPs showed in **(A)**. The initial significant differences between ratios were abolished during the hypobaric exposure, and recovered 24 h after it. Values are expressed as mean ± SEM. Statistical differences, ^∗^*P* < 0.05; ^∗∗^*P* < 0.01. Student *t*-test.

We further checked the evolution of paired-pulse facilitation, before, 15 and 60 min after the beginning, and 24 h after exposure to hypobaric conditions (**Figure 3A**). The facilitation evoked by the presentation of a pair of pulses is a typical presynaptic short-term plastic property of excitatory hippocampal synapses and it has been correlated with neurotransmitter release (Zucker and Regehr, 2002). In accordance, it is expected that, at the CA3–CA1 synapse, animals present a larger fEPSP response to the second stimulus (with respect to the first) at short intervals (≤100 ms). Thus, maximum amplitudes to the second pulse were reached at 40 ms of interstimulus interval for control animals (0.61 ± 0.09 mV), while GYS1^Nestin-KO^ did it at 100 ms (0.91 ± 0.1 mV). GYS1^Nestin-KO^ mice presented differences with the amplitudes evoked by the first pulse at 10, 40, and 100 ms of interstimulus intervals. In hypobaric conditions, the amplitude of fEPSPs produced by the first pulse was higher in both groups of mice. This increase with respect to the initial situation was significant (*P* < 0.001, *t*-Test) for fEPSPs evoked by the first pulse for the GYS1^Nestin-KO^ group 60 min after the start of the hypoxia (**Figure 3A**; 5000 m, 60 min), reaching values of 0.79 ± 0.09 mV, while at ground levels reached 0.48 ± 0.06 mV. At ground level, the GYS1^Nestin-KO^ group presented a significantly larger facilitation to the paired-pulse test than controls. Under hypoxic conditions, these differences were abolished, although again they were partially recovered 24 h after the hypobaric episode. In the same regard, the St2/St1 ratios were significantly different before exposure to hypoxia, between 10 and 100 ms of interstimulus interval, when the GYS1^Nestin-KO^ group reached St2/St1 ratios of 2.1 ± 0.2 at 40 ms of interstimulus interval, while controls did 1.3 ± 0.1. These differences were abolished during the hypoxic episode, and recovered 2-4 h later, reaching the GYS1^Nestin-KO^ group ratios of 1.9 ± 0.2, while controls did 1.3 ± 0.1 (**Figure 3B**).

### Effects of Kainate Injection

**FIGURE 4:**
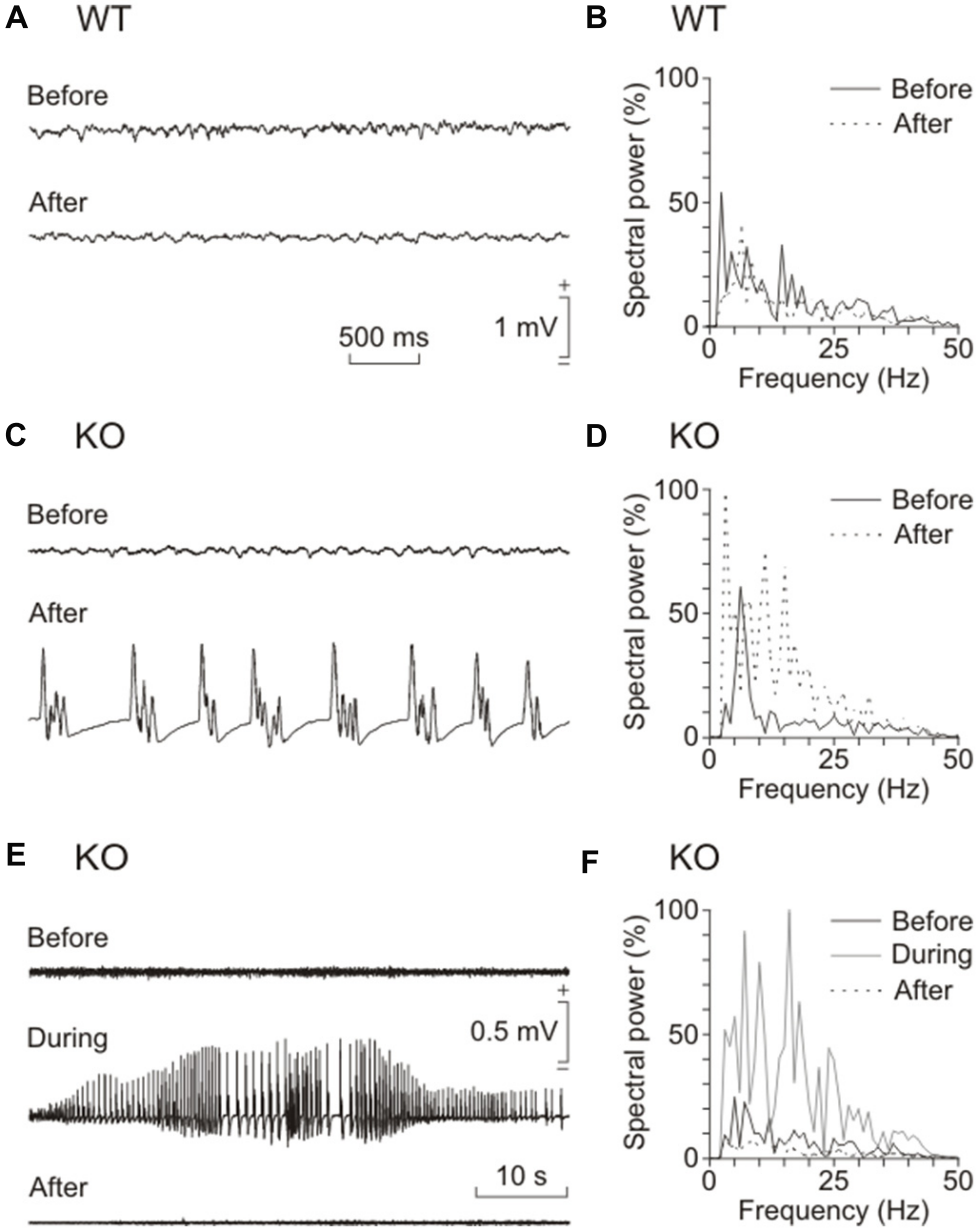
**Effects of kainate injection on spontaneous LFPs recorded from the two groups of mice. (A)** Representative examples of LFPs recorded from a wild-type (WT) mouse before and 30 min after a kainate injection (8 mg/kg, i.p.). **(B)** Spectral analysis of hippocampal LFP recordings collected from a representative WT mouse before (continuous trace) and after (dotted trace) kainic injection. **(C,D)** Same set of data collected from a representative GYS1^Nestin-KO^ (KO) mouse. Note that the presence of repetitive (≈2–3 Hz) clonic seizures **(C)** significantly (*P* < 0.05) increased the amplitude of the power spectrum (dotted line in **D**). **(E,F)** LFP recordings **(E)** and spectral power analysis **(F)** of a tonic seizure evoked in a KO animal by the kainate injection. Note that the long-lasting seizure (dotted line in **F**) canceled out (before vs. after, *P* < 0.001) the normal theta rhythm (black line in **F**) present in hippocampal LFP recordings. Also note that the spectral power increased significantly (*P* < 0.001) in different (5–10 and 10–15 Hz) frequency bands during the seizure (gray line in **F**). One-way ANOVA.

**FIGURE 5:**
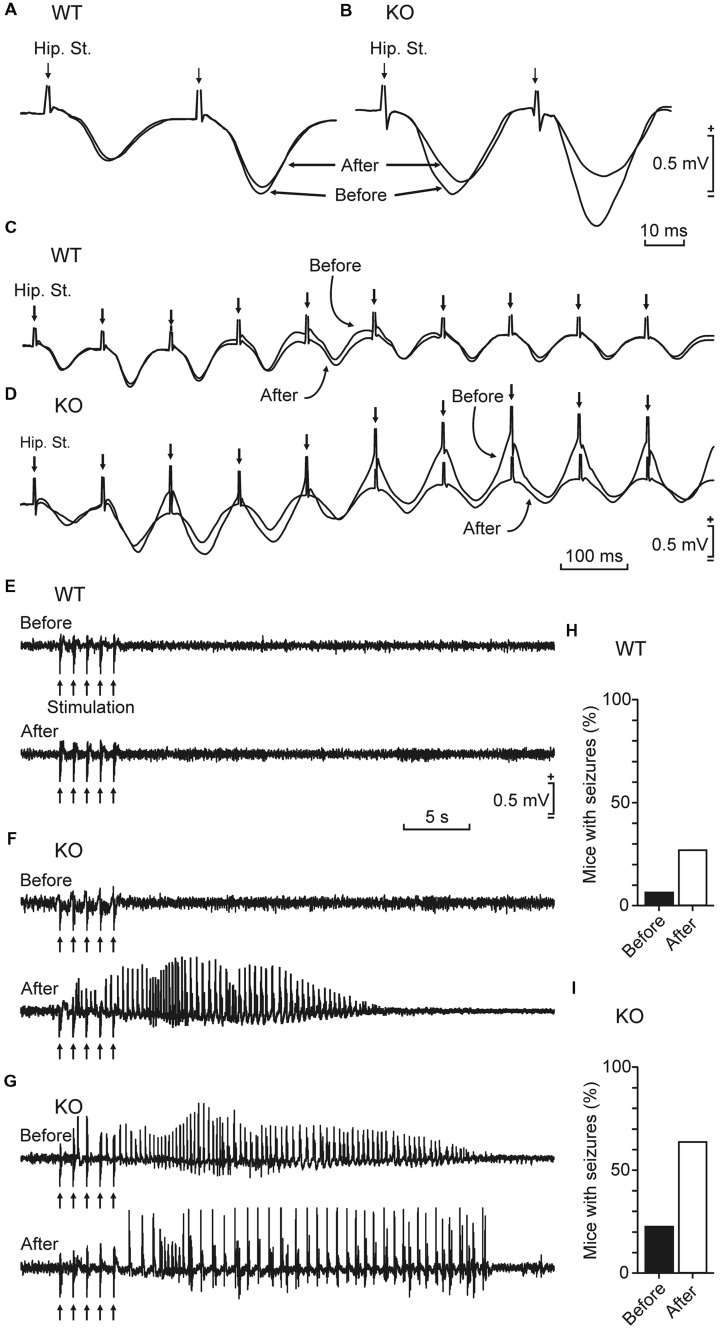
**Effects of kainate injection and train stimulation in GYS1^Nestin-KO^ behaving mice. (A,B)** Representative fEPSPs evoked at the hippocampal CA3–CA1 synapse of a wild-type (**A**, WT) and a GYS1^Nestin-KO^ (**B**, KO) animal before and after kainate injection (8 mg/kg, i.p.). Note that the presence of clonic seizures significantly reduced the amplitude of the evoked fEPSP in the KO mouse. **(C,D)** Effects on fEPSPs of train stimulation (10 Hz) of the hippocampal CA3–CA1 synapse in a wild-type (**C**, WT) and a *GYS1*^Nestin-KO^ (**D**, KO) animal during tonic-clonic seizures. Records were collected before and after a kainate injection that evoked seizures only in the KO mouse. Note that the presence of spontaneous seizures evoked by kainate injection decreased the amplitude of fEPSPs evoked by train stimulation. **(E–G)** Differential effects of train stimulation (arrows: five 200 Hz, 100 ms trains of pulses at a rate of 1/s) of WT and KO mice before and after kainate injection. Note the long-lasting seizure evoked in the KO animal even before kainate injection **(G)**. In all cases, intensity of train stimulation was set at ≈40% of that necessary to evoke a maximum response during the input/output test. **(H,I)** Percentage (%) of WT (*n* = 16) and KO (*n* = 14) mice presenting seizures following train stimulation before **(H)** and after **(I)** kainate injection.

Kainate injection is a widely used protocol to induce epilepsy in mice (McKhann et al., 2003). To determine the relevance of brain glycogen in epilepsy, we tested the susceptibility of control and GYS1^Nestin-KO^ mice to a single (8 mg/kg, i.p.) injection of kainate. Our results showed a noticeable difference in susceptibility to spontaneous seizures and in their intensity. **Figure 4** illustrates representative examples of LFPs recorded from the two groups. The GYS1^Nestin-KO^ group showed the highest number of hippocampal seizures, accompanied on occasions by myoclonus (**Figures 4C,D**). The spectral power of the recorded LFPs increased significantly (*P* < 0.001) in different (5–10 and 10–15 Hz) frequency bands during the seizures (gray line in **Figure 4F**). Interestingly, kainate-induced seizures produced a significant (*P* < 0.001) decrease in the power spectra of the theta-band component of the LFP recorded in the CA1 area of GYS1^Nestin-KO^ mice (**Figures 4E,F**). We also analyzed fEPSP at the hippocampal CA3–CA1 synapse in the two groups of mice before and after kainate injection (**Figures 5A,B**). The treatment produced a decrease in paired-pulse facilitation (**Figure 5B**) and in the response of the CA3–CA1 synapse to train (10 Hz) stimulation (**Figure 5D**). This result was not observed in control animals (**Figures 5A,C**). Finally, train stimulation of Schaffer collaterals evoked more frequent and longer lasting after-discharges in GYS1^Nestin-KO^ mice than in control counterparts (21.4% vs. 6.25% before, and 64.2% vs. 25% after kainate injection; **Figures 5E–I**). On the whole, GYS1^Nestin-KO^ mice presented a greater propensity than controls to generate hippocampal seizures, mostly when activated by kainate injection or by train stimulation of Schaffer collaterals.

## Discussion

Studying the function of brain glycogen is greatly complicated by the lability of this polysaccharide during sample extraction and measurement, the complexity of its regulation, and the lack of sensitive *in vivo* measurement methods. In this scenario, our brain-specific GS KO is a valuable tool (Duran et al., 2013) that allows the analysis of the consequences of the absence of glycogen in the brain. We previously described impaired memory formation and synaptic plasticity in GYS1^Nestin-KO^ animals (Duran et al., 2013), thereby confirming that brain glycogen participates in the normal functioning of the brain in physiological conditions and that it plays a key role in memory acquisition and learning. Here we took advantage of this animal model to address the role of brain glycogen in response to hypoxia and its involvement in susceptibility to epilepsy. Our results confirm the importance of this polysaccharide in both energetically challenging conditions.

Brain glycogen has traditionally been considered an energy reservoir for certain stresses in which energy supply to the brain is impaired, such as in hypoxia, ischemia, and hypoglycemia. For this reason, we analyzed the consequences of the lack of glycogen in the brain under exposure to hypobaric conditions, a widely used technique to induce hypoxia in animal models (Guerra-Narbona et al., 2013) and in which, contrary to other models such as ischemia, the availability of glucose is not compromised. When exposed to hypobaric hypoxia, synaptic excitability was increased in control and GYS1^Nestin-KO^ animals, as determined by a leftward displacement of input/output curves, although the increased paired-pulse facilitation observed in GYS1^Nestin-KO^ mice was abolished. There is therefore an excitability factor in the CA3–CA1 synapse during hypoxia, which may be due to the increase in glutamate concentration, as described previously (Ye et al., 2010). Interestingly, this effect is partially prevented by the hypoxia inducible factor (HIF; Badawi et al., 2012), responsible for the expression of genes encoding various glycolytic enzymes and for glucose and lactate transporters (Brix et al., 2012). This excitability was noticeable at higher intensities only in control mice, probably because of the energetic deficits in GYS1^Nestin-KO^ mice. Thus, hypoxia produced different effects on paired-pulse facilitation in GYS1^Nestin-KO^ with respect to controls, thereby suggesting a differential activation of presynaptic mechanisms (Madroñal et al., 2009) in these genetically manipulated mice (Duran et al., 2013). This mechanism may serve to compensate for the unavailability of glycogen through the facilitation of a less metabolically dependent presynaptic neurotransmitter release (Duran et al., 2013; Sáez et al., 2014) while energy availability is not a limiting factor.

Several studies have proposed a relationship between brain glycogen and susceptibility to epilepsy (Phelps, 1975; Hevor et al., 1985; Bernard-Helary et al., 2000; Dalsgaard et al., 2007). In this regard, here we analyzed the susceptibility of GYS1^Nestin-KO^ animals to kainate-induced epilepsy. Indeed, these animals presented a greater susceptibility to hippocampal seizures following kainate administration (Duran et al., 2013). Furthermore, the intensity of the seizures was higher in the GYS1^Nestin-KO^ animals than in their littermate controls. In this regard it is important to point out that GYS1^Nestin-KO^ animals presented a larger disposition to synchronize their LFPs (mostly for 6-10 Hz, i.e., the high theta band) under hypoxic conditions that their littermate controls. As a whole, these results indicate that glycogen availability contributes to the maintenance of the proper equilibrium between excitatory and inhibitory neurotransmission. This importance might rely on the role of astrocytic glycogen in the uptake of potassium and glutamate from the synaptic cleft. Alternatively, our results could be attributed to inhibitory neurons firing at very high frequency, which implies high energy expenditure to maintain membrane potential. The lack of glycogen could result in a deficient inhibition, thus increasing susceptibility to epileptic seizures.

Our results demonstrate the importance of brain glycogen in energetically challenging conditions. This importance might be explained by the fact that glycogen breakdown to glucose-6-phosphate is more rapid than the incorporation and phosphorylation of extracellular glucose (Shulman et al., 2001). Accordingly, glycogen degradation in the brain would be essential for the rapid generation of energy, even in euglycemic conditions. As we have previously demonstrated that neurons contain a small but functionally important amount of glycogen (Sáez et al., 2014), the results presented here could also be, in part, attributable to the glycogen present in neurons, since glycogen synthase in these cells is also knocked out in GYS1^Nestin-KO^ mice.

## Conflict of Interest Statement

The authors declare that the research was conducted in the absence of any commercial or financial relationships that could be construed as a potential conflict of interest.
